# Extensively Drug-Resistant Tuberculosis, Pakistan

**DOI:** 10.3201/eid1609.100280

**Published:** 2010-09

**Authors:** Rumina Hasan, Kauser Jabeen, Asho Ali, Yasraba Rafiq, Rabia Laiq, Babar Malik, Mahnaz Tanveer, Ramona Groenheit, Solomon Ghebremichael, Sven Hoffner, Zahra Hasan

**Affiliations:** Author affiliations: Aga Khan University, Karachi, Pakistan (R. Hasan, K. Jabeen, A. Ali, Y. Rafiq, R. Laiq, B. Malik, M. Tanveer, Z. Hasan);; Swedish Institute for Infectious Disease Control, Solna, Sweden (R. Groenheit, S. Ghebremichael, S. Hoffner)

**Keywords:** Bacteria, tuberculosis and other mycobacteria, drug resistance, Pakistan, XDR TB, multidrug resistant, extensively drug-resistant tuberculosis, genotype, Beijing strains, dispatch

## Abstract

Frequency of extensively drug-resistant tuberculosis in Pakistan increased from 1.5% in 2006 to 4.5% in 2009 (p<0.01). To understand the epidemiology, we genotyped selected strains by using spoligotyping, mycobacterial interspersed repetitive units–variable number of tandem repeats, and IS*6110* restriction fragment length polymorphism analysis.

Emergence and spread of multidrug-resistant (MDR) and extensively drug-resistant (XDR) tuberculosis (TB) are facilitated by inadequate detection and treatment (*1*). TB detection and treatment are more difficult in countries, like Pakistan, that are facing complex emergencies, including humanitarian crises and conflicts (*2*). Published data report an increasing prevalence of MDR TB and emergence of XDR TB strains in Pakistan (*3*).

This study documents an increasing trend of XDR resistance within MDR TB isolates in Pakistan. To obtain better insight into the epidemiology of these strains, we genotyped selected XDR TB strains by using spoligotyping, mycobacterial interspersed repetitive units–variable number of tandem repeats (MIRU-VNTR), and IS*6110* restriction fragment length polymorphism (RFLP) analysis. This study was approved by the Ethics Review Committee of the Aga Khan University and conducted at the Aga Khan University Hospital, Karachi, Pakistan.

## The Study

The Aga Khan University Hospital laboratory receives specimens from >180 collection units located in major cities and towns in Pakistan. Specimens for TB cultures are requested by physicians as required and received through passive specimen collection. Past treatment history is usually not available and thus could not be included in this study. *Mycobacterium tuberculosis* was isolated from the specimens by using Lowenstein-Jensen and MGIT (Becton Dickinson, Franklin Lakes, NJ, USA) media. It was then identified by using the BACTEC NAP TB differentiation test (Becton Dickinson), growth in para-nitrobenzoic acid–containing media, nitrate reduction, and niacin accumulation (*4*).

We performed susceptibility testing by using an agar proportion method on enriched Middlebrook 7H10 medium (BBL Microbiology Systems, Cockeysville, MD, USA) at the following concentrations: rifampicin 1 μg/mL, isoniazid 0.2 μg/mL, streptomycin 2 μg/mL and 10 μg/mL, and ethambutol 5 μg/mL. Pyrazinamide sensitivity was determined by using BACTEC 7H12 medium, pH 6.0, at 100 μg/mL (BACTEC PZA test medium, Becton Dickinson). MDR TB strains were further tested with capreomycin 10 μg/mL, ciprofloxacin 2 μg/mL, ethionamide 5 μg/mL, amikacin 5μg/mL, and kanamycin 6 μg/mL. Reference strain *M. tuberculosis* H37Rv was used as a control with each susceptibility testing batch (*5*). *M. tuberculosis* drug-susceptibility testing is validated by the Supranational Laboratory Network of the World Health Organization (www.who.int/drugresistance/tb/en/).

MDR TB was defined as resistance to at least isoniazid and rifampicin. XDR TB was defined as resistance to quinolones and to 1 of the injectable second-line drugs in addition to MDR. Randomly selected isolates were confirmed as XDR TB by using the GenoType MTBDRsl assay (HAIN Lifesciences, Nehren, Germany). XDR TB isolates were stored at –80°C. Stored XDR TB strains were revived for genotyping; 57 isolates that grew were used for spoligotyping and MIRU-VNTR typing (3 isolates from 2006, 5 from 2007, 19 from 2008, and 30 from 2009).

DNA was extracted by using the cetyltrimethylammonium bromide method (*6*). Spoligotyping was performed by using a commercially available kit provided by Isogen Life Science (De Meern, the Netherlands). Spoligotyping based on the 43 spacers of the DR region of *M. tuberculosis* complex was performed by using primers DRa 5′-GGTTTTGGGTCTGACGAC-3′ and DRb 5′-CCGGAGAGGGGACGGAAAC-3′ as described (*7*). Negative and positive controls, including template-free PCR–amplified reaction mixture and *M. tuberculosis* H37Rv DNA, were used with each spoligotype blot.

Data extracted from the computerized information system of the hospital were transferred to the statistical software SPSS version 14.0 (SPSS, Chicago, IL, USA). Frequencies with percentages were computed for each year. We used χ^2^ for trend analysis to assess resistance trends over the study period. A p value <5% was considered significant.

The spoligotyping results were entered in the BioNumerics Software version 3.5 (Applied Maths Program, Biosystematica, Ceredigion, UK). A dendrogram was generated by using the unweighted pair–group method with arithmetic averages calculation. The spoligotypes were compared with the most prevalent *M. tuberculosis* subfamilies as identified by the World Spoligotyping Database SpolDB4.0 of Pasteur Institute of Guadeloupe (www.pasteur-guadeloupe.fr/tb/bd_myco.html) (*8*), which includes >40,000 isolates split into 1,030 shared types and >3,530 orphan profiles.

The isolates were genotyped by PCR amplification of 15 MIRU-VNTR loci by using standard methods as described (*9*). Sizes of the PCR fragments and assignment of the various VNTR alleles were also determined by using standard protocol for gel electrophoresis (www.genoscreen.com). All reactions were performed in duplicate by using standard positive and negative controls supplied in the MIRU-VNTR validation kit.

IS*6110* RFLP of *M. tuberculosis* strains were performed by standardized methods (*10*). Briefly, XDR TB strains were recovered on LJ medium. DNA was extracted from the strains by standard methods (*6**,**10*). *Pvu*II-digested DNA was subjected to agarose gel electrophoresis and Southern blotting. DNA fingerprinting was performed by hybridization with the IS*6110* by using the enhanced chemiluminescence method (Amersham Biosciences, Piscataway, NJ, USA).

During 2006–2009, a total of 9,523 *M. tuberculosis* strains were isolated, including 3,682 (38.7%) MDR TB strains. Although the MDR TB rate remained constant (Table 1), the XDR TB rate (expressed as a percentage of MDR TB isolated in a year) showed a significant increase. The XDR TB strains were from specimens received from 23 cities; the largest numbers were from Karachi (22), Hyderabad (13), and Peshawar (12). Mean ± SD age of patients with XDR TB was 37 ± 14 (range 16–80) years; 57.15% were men and 42.85% women.

**Table 1:** Frequency of multidrug-resistance and extensively drug-resistance among *Mycobacterium tuberculosis* isolates, Pakistan, 2006–2009*

Year	No. *M. tuberculosis* isolates	MDR isolates, no. (%)	MDR + ciprofloxacin-resistant isolates, no. (% of total MDR)	Total no. XDR isolates	XDR as % of total MDR
2006	1,917	728 (38)	131 (18)	11	1.5
2007	2,019	782 (39)	168 (21.5)	17	2.2
2008	2,584	991 (38)	356 (36)	32	3.2
2009	3,003	1,181 (39)	512 (43)	53†	4.5

*MDR, multidrug resistant; XDR, extensively drug resistant. †By trend analysis (p value for trend <0.01).

Diversity of XDR TB strains among the 57 isolates genotyped is shown by spoligotyping (Table 2) and MIRU-VNTR analysis. Spoligotyping data points to the predominance of strains belonging to the Central Asian Strain (CAS) 1 family (n = 24 [42.1%]). Overall CAS strains included CAS1_KILI ST21 (n = 2); CAS1_DEHLI ST25 (n = 1); CAS1, ST26 (n = 24); CAS1_DEHLI ST428 (n = 1); CAS1_DEHLI ST794 (n = 1); CAS1_DEHLI ST1198 (n = 3); and CAS1_DEHLI ST1401 (n = 1). The second largest family comprised the Beijing (ST1) genogroup (n = 5 [8.8%]). Four (7%) strains of T family (ST53 and ST804) and 2 strains of U family (ST346) were also identified. Additional XDR TB strains included 1 (2%) strain each of EAI (ST11) and X (ST200). Furthermore, 1 new unmatched cluster and 9 unmatched orphan types were also recognized. MIRU-VNTR patterns of all XDR TB strains were different, indicating a lack of clustering among the strains studied. Randomly selected strains present in CAS1 (ST26, n = 6) and Beijing (ST1, n = 3) clusters were subjected to IS*6110* RFLP typing. RFLP patterns of each of these strains also differed, further confirming a lack of clustering among the XDR TB strains studied.

**Table 2:** Spoligotype distribution of 57 extensively drug-resistant strains of *Mycobacterium tuberculosis*, Pakistan, 2006–2009*

Genogroup and shared type	No. (%) isolates	Overall %
CAS		57.9
ST21	2 (3.5)	
ST25	1 (1.8)	
ST26	24 (42.1)	
ST428	1 (1.8)	
ST794	1 (1.8)	
ST1198	3 (5.3)	
ST1401	1 (1.8)	
Beijing		8.8
ST1	5 (8.8)	
EAI3		1.8
ST11	1 (1.8)	
U		1.8
ST346	2 (3.5)	
T1		7.0
ST53	1 (1.8)	
ST804	3 (5.3)	
X3		1.8
ST200	1 (1.8)	
Unmatched cluster	2 (3.5)	3.5
Orphan types	9 (15.8)	15.8

*ST, spoligotype; CAS, Central Asian strain; EAI, East African Indian strain.

## Conclusions

This study demonstrated a rising XDR-TB trend in Pakistan and raises concerns despite the fact that Pakistan’s 2009 XDR rate (4.5%) of MDR TB is below the global average (6.6%–23.7%) (*11*). Genotyping data are comparable with those from earlier studies (*12**,**13*), suggesting dominance of CAS1 strains. The fact that Beijing family strains were 9% of XDR TB isolates vs. 3% prevalence in the overall MDR TB reported in this population (*13*) suggests that Beijing strains are associated with drug resistance in Pakistan and adjacent countries (*14*).

Furthermore, the finding that the XDR TB strains in our study were genetically diverse argues against dissemination of 1 particular genogroup responsible for drug resistance and supports the concept that XDR TB in Pakistanis is likely to be a consequence of inadequate treatment of TB. The challenging sociopolitical situation in Pakistan is likely to exacerbate this public health problem. Emergency measures are required to avoid an exponential rise in drug-resistant TB in the country and the region. We recommend that increased XDR TB rates in this area be considered not just of national concern but also be recognized as a regional public health issue requiring introduction of cooperative and support measures aimed at limiting the spread of drug-resistant TB within southern Asia.
